# Chemoradiotherapy and transanal endoscopic surgery versus transanal endoscopic surgery alone in T1N0M0 rectal cancer: a multicenter, randomized, controlled, phase III clinical trial (TAUTEM-T1 study)

**DOI:** 10.1007/s00384-026-05112-y

**Published:** 2026-04-23

**Authors:** Xavier Serra-Aracil, Anna Nonell, Cristina Gener-Jorge, Carles Pericay, Thomas Golda, Esther Kreisler, Eloy Espin-Basany, Anna Pallisera, Jesus Badia-Closa, Beatriz Espina, Nerea Borda-Arrizabalaga, Angel Reina, Hector Guadalajara-Labajo, Ana Otero, Salvadora Delgado, Miquel Kraft, Rosa Querol, Blas Flor, Gianluca Pellino, Sebastiano Biondo, Aleidis Caro-Tarrago

**Affiliations:** 1 Department of Surgery, Universitat Autònoma de Barcelona, Coloproctology Unit, Parc Tauli University Hospital, Institut d’investigació I Innovació Parc Tauli I3PT-CERCA, Sabadell (Barcelona), Spain; 2 Department of Pathology, Universitat Autònoma de Barcelona, Parc Tauli University Hospital, Institut d’investigació I Innovació Parc Tauli I3PT-CERCA, Sabadell (Barcelona), Spain; 3 Medical Oncology Department, Mutua de Terrassa University Hospital, Terrassa (Barcelona), Spain; 4 Colorectal Unit, General and Digestive Surgery Department, Bellvitge University Hospital, Barcelona, Spain; 5 Colorectal Unit, General and Digestive Surgery Department, Vall d’Hebron University Hospital, Departamento de Cirugía, Universitat Autònoma de Barcelona, Barcelona, Spain; 6 Colorectal Unit, General and Digestive Surgery Department, Hospital de San Juan Despí Moisès Broggi, Barcelona, Spain; 7 Colorectal Unit, General and Digestive Surgery Department, Santa Creu I Sant Pau University Hospital, Barcelona, Spain; 8 General and Digestive Surgery Department, Donostia University Hospital, Donostia, Gipuzkoa Spain; 9 Colorectal Unit, Unidad de Gestión Clínica Cirugía y Area de Gestión Norte de Almería, Complejo Hospitalario Torrecárdenas, Almería, Spain; 10 Colorectal Unit, General and Digestive Surgery Department, Hospital Universitario Fundación Jiménez Díaz, Madrid, Spain; 11 General and Digestive Surgery Department, Hospital Clinic University Hospital, Barcelona, Spain; 12 Colorectal Unit, General and Digestive Surgery Department, Mutua de Terrassa University Hospital, Terrassa (Barcelona), Spain; 13 Department of Medical Oncology, Universitat Autònoma de Barcelona, Coloproctology Unit, Parc Tauli University Hospital, Institut d’investigació I Innovació Parc Tauli I3PT-CERCA, Sabadell, Spain; 14 Colorectal Unit, General and Digestive Surgery Department, La Fe University Hospital, Valencia, Spain; 15 Colorectal Unit, General and Digestive Surgery Department, Joan XXIII University Hospital, Tarragona, Spain

**Keywords:** Rectal cancer, Neoadjuvant treatment and rectal cancer, Local excision and rectal cancer, Transanal endoscopic microsurgery (TEM), Total mesorectal excision (TME)

## Abstract

**Purpose:**

For clinical (c) T1N0M0 rectal adenocarcinoma without adverse pathological features, local excision by transanal endoscopic surgery (TEM) is standard; however, contemporary series still report local recurrence (LR) rates of 15–20%. Preoperative unfavorable histopathologic features cannot reliably identify high-risk pT1 disease, and both completion total mesorectal excision (TME) and salvage TME for LR carry relevant morbidity and functional impairment. Building on our prior phase III trial in T2–T3abN0M0 (TAUTEM study), showing that preoperative chemoradiotherapy (CRT) followed by TEM achieved a 7.4% LR with improved postoperative outcomes, we hypothesize that CRT + TEM will increase rectal preservation in cT1N0M0 without compromising oncologic safety or quality of life. The TAUTEM-T1 trial tests this hypothesis.

**Methods:**

Multicenter, prospective, randomized, controlled, phase III superiority trial. Adults with biopsy-proven rectal low or moderate grade adenocarcinoma ≤ 4 cm, located < 10 cm from the anal verge, staged as cT1N0M0, are randomized (1:1) to: CRT (long-course radiotherapy with concurrent capecitabine) followed by TEM at week 10, or TEM alone. The primary endpoint is rectal preservation at 3 years. Secondary endpoints include postoperative morbidity/mortality, CRT-related adverse events, quality of life and anorectal function, and long-term oncologic outcomes (local/distant recurrence, overall and disease-free survival). Planned sample size: 106 patients.

**Results:**

This manuscript describes the rationale and design of the TAUTEM-T1 randomized trial.

**Conclusion:**

TAUTEM-T1 will assess whether preoperative CRT followed by TEM increases rectal preservation in cT1N0M0 rectal cancer without compromising oncologic safety, quality of life, or bowel function.

**Trial registration:**

ClinicalTrials.gov, NCT06450574.

## Introduction

Management of early rectal cancer (stage I: T1–T2, N0, M0) varies widely. For T1N0M0 tumors without adverse pathological factors, local excision (LE) is the standard treatment per international guidelines [1, 2]. However, in T1 lesions with adverse features and in T2 tumors, lymph node involvement may reach 12–28%, warranting total mesorectal excision (TME) [3].

TME, described by Heald, markedly reduced local and systemic recurrence in rectal cancer [4]. However, it is associated with substantial morbidity, including temporary/permanent stomas and relevant mortality, as well as long-term functional sequelae such as genitourinary dysfunction and low anterior resection syndrome (LARS) [5, 6]. In appropriately selected patients, transanal endoscopic local excision (LE) preserves the rectum and is associated with lower morbidity and minimal sphincter/genitourinary impairment [7, 8]. Nonetheless, despite a generally favorable prognosis in stage I disease, standard management does not typically include neoadjuvant therapy [9].

pT1 rectal adenocarcinomas can be classified into two groups according to surgical and histological criteria: (1) favorable prognosis tumors, with a local recurrence risk < 5%; and (2) unfavorable prognosis tumors, with a local recurrence risk up to 29% [10]. Favorable surgical factors include en bloc resection, full-thickness excision, and clear margins > 1 mm. Unfavorable histopathologic factors associated with nodal involvement include depth of submucosal invasion, poor differentiation, venous/lymphatic or perineural invasion, positive margins (< 1 mm), and tumor budding [2, 8]. Depth of submucosal invasion is the main determinant of nodal risk [3], as most other adverse factors occur in < 10% of pT1 cases [1]. Its prognostic value has been questioned by the meta-analysis by Zwager et al. [12], although several included studies had methodological limitations.

There are several classifications used to assess submucosal invasion (Haggitt, Kikuchi, Ueno, Kudo, and Kitajima); however, they present limitations and relevant variability that may influence clinical decision-making. Therefore, our group has proposed the Taulí-pT1 classification, which quantifies tumor invasion according to the area of residual healthy submucosa in the specimen, using a uniform methodology based on a percentage derived from a constant reference point [11]. This classification shows excellent correlation with Kitajima’s and very good correlation with Kudo’s system. Its only requirement for applicability is the presence of the muscularis propria in the specimen. Recently, we have published its validation through interobserver concordance analysis [13].

Recent studies with large cohorts and long-term follow-up (although with retrospective designs) have reopened the debate on local surgery for pT1N0M0 rectal cancer. Leijtens et al. [14] reported, in a series of 150 patients with a median follow-up of 58.9 months, a local recurrence (LR) rate of 22.7% and an overall survival (OS) of 82%; even when stratifying tumors without adverse prognostic features, LR rates exceed 20%. Recently, we have published our long-term follow-up cohort, with a median follow-up of 91 months (IQR: 84), 17 patients (16.3%) developed LR and 14 (13.5%) developed distant recurrence. Five-year rectal cancer–specific and overall survival rates were 95% and 74%, respectively. Among the 88 patients without histopathological or surgical high-risk factors, 13 (14.8%) experienced LR and 9 (10.2%) distant recurrence [15].

In patients with locally advanced rectal cancer, preoperative chemoradiotherapy (CRT) prior to TME has reduced local recurrence and improved survival [16]. This approach achieves partial pathological response (pPR) in approximately 54–75% of cases and complete pathological response (pCR) in 8–27% [17]. The possibility of tumor disappearance after CRT has encouraged rectal preservation strategies, thereby avoiding the undesirable effects of TME. Habr-Gama et al. [18] reported that, in patients with a complete clinical response (cCR) after CRT, close surveillance could avoid any surgery, allowing rectal preservation (the “watch and wait” strategy, WW). However, preoperative identification of cCR remains challenging, as it does not always correlate with cPR [19, 20]. The main limitation of this strategy is the local regrowth rate, reported to be as high as 38%, although overall survival (OS) and disease-free survival (DFS) outcomes have been satisfactory [21].

Our group (TAUTEM) recently published the results of a multicenter, randomized phase III trial in T2–T3abN0M0 rectal cancer comparing standard TME with preoperative CRT followed by LE(CRT-TEM) [22]. The experimental CRT-TEM arm achieved a pCR rate of 44.3%. With a 5-year follow-up (intention-to-treat analysis, ITT), LR was 6.2% for TME versus 7.4% for CRT-TEM (difference − 1.23; 95% CI − 8.98 to 6.51), meeting the non-inferiority criterion (primary endpoint). Distant recurrence (DR) rates were 17.3% (TME) versus 12.3% (CRT-TEM) (difference 4.94; 95% CI − 5.98 to 15.85), and OS was 85.2% (TME) versus 82.7% (CRT-TEM) (difference 2.47; 95% CI 0.38 to 1.78).

An initial proposal of our group when designing this protocol was to apply preoperative CRT followed by TEM in pT1N0M0 rectal tumors with adverse prognostic factors. The main limitation, however, is that these factors can only be identified after the pathological examination of the LE specimen. Preoperative biopsy merely confirms that the lesion is an infiltrating adenocarcinoma, and the only identifiable adverse feature is poor differentiation, present in less than 10% of cases [11, 14]. Therefore, it is not possible to reliably identify high-risk pT1N0M0 patients preoperatively. Moreover, as previously mentioned, in pT1N0M0 tumors without adverse features, LR exceeds 15–20% [11, 14], more than twice that observed in pT2–3abN0M0 cases from the TAUTEM study treated with neoadjuvant therapy and TEM (LR 7.4%) [22]. Hence, our hypothesis is to treat clinically staged T1N0M0 (cT1N0M0) rectal adenocarcinomas with preoperative CRT to reduce LR below that observed in the TAUTEM trial and increase rectal preservation [22, 23].

Primary objective. To evaluate whether preoperative CRT followed by transanal endoscopic microsurgery (CRT–TEM) increases rectal preservation at 3 years in patients with T1N0M0 rectal cancer without compromising oncologic outcomes, taking into account that local recurrences will be managed with TME, and that, after pathological examination of the specimen, cases presenting surgical or pathological adverse features or with upstaging to ≥ pT2 will also require completion TME according to protocol. Therefore, the challenge is to minimize the number of TMEs, given their well-documented surgical morbidity and functional impairment.

## Hypothesis and objectives

### Hypothesis

In patients with rectal adenocarcinoma (with biopsy-proven rectal low or moderate grade adenocarcinoma) staged as cT1N0M0 (≤ 4 cm in size and < 10 cm from the anal verge) by endorectal ultrasound (ER) and pelvic magnetic resonance imaging (MRI), preoperative chemoradiotherapy followed by LE(CRT–TEM) is superior to standard LE (TEM) in terms of rectal preservation rate (primary endpoint). Furthermore, CRT–TEM is expected to reduce LR, DR and improve OS, rectal cancer–specific survival, and DFS (at 3 years), without negatively affecting postoperative morbidity or quality of life.

## Objectives

### Primary objective

To compare the rectal preservation rate between TEM and CRT–TEM at 3 years after surgery.

### Secondary objectives

To assess the tolerance and adverse effects (acute and chronic) of preoperative chemoradiotherapy (CRT) in the CRT–TEM group (patient safety). To analyze postoperative morbidity and mortality in both groups (surgical outcomes). To evaluate clinical and pathological responses to CRT. To assess quality of life at 1 year after surgery in both groups. To compare long-term oncologic outcomes between groups (at 3 years): LR, DR, DFS, OS and rectal cancer–specific survival at 3 years.

## Methods

### Study design

Phase III, multicenter, prospective, controlled, randomized superiority clinical trial evaluating the efficacy and safety of preoperative CRT followed by LE (CRT-TEM group) versus LE alone (TEM group) in clinically staged cT1N0M0 rectal cancer, with rectal preservation as the primary outcome.

### Patients – study population

Patients diagnosed with rectal tumor will undergo full colonoscopy with multifocal biopsy, which will describe the tumor size and its distance from the anal verge. Staging will include endorectal ultrasound (ER) [24], pelvic magnetic resonance imaging (MRI), and abdominal and chest computed tomography (CT). Baseline serum tumor markers, carcinoembryonic antigen (CEA) and carbohydrate antigen 19–9 (CA 19–9), will also be determined.

### Inclusion criteria

Indication for local excision by a multidisciplinary committee according to ESMO and NCCN guidelines [1, 2] in patients with pathologically confirmed rectal adenocarcinoma on biopsy (with biopsy-proven rectal low or moderate grade adenocarcinoma), located ≤ 10 cm from the anal verge as measured by rigid rectoscopy at the time of endorectal ultrasound (ER). Age ≥ 18 years (both men and women). Women of childbearing potential must have a negative urine pregnancy test at the screening visit. Preoperative staging by ER and/or MRI must confirm T1N0 disease; in the event of discrepancy, the higher category will be adopted as the preoperative diagnosis. Tumor size must be ≤ 4 cm (maximum diameter on MRI). cN0 is defined as absence of suspicious lymph nodes on ERUS/EUS and pelvic MRI; if nodal involvement is suspected on either modality, the patient is considered cN + (higher category) and is not eligible as cN0. ASA score ≤ III. Absence of distant metastases on abdominopelvic CT and chest X-ray (or chest CT if inconclusive). Written informed consent must be obtained before performing any study-specific procedure.

Endorectal ultrasound (ER) staging will follow established criteria as previously described by Hildebrandt [25]. Preoperative pelvic MRI staging will be performed according to standard high-resolution rectal cancer MRI protocols [26, 27]. Tumor size (maximum diameter in mm) will be recorded as a baseline morphological parameter but will not be used as a sole criterion for response assessment in patients undergoing neoadjuvant therapy.

### Exclusion criteria

Preoperative staging by ER or pelvic MRI showing disease beyond T1 or N0 stage; presence of distant metastases; synchronous colorectal adenocarcinomas; poorly differentiated rectal adenocarcinoma [10]; contraindication and/or intolerance to preoperative chemotherapy or radiotherapy.

Unfavorable histopathologic features such as lymphovascular invasion, perineural invasion, and tumor budding cannot be reliably assessed on baseline endoscopic biopsy specimens; therefore, at screening, only tumor differentiation grade is used for eligibility, whereas adverse features are evaluated on the local excision specimen [28].

Patients not eligible for inclusion in the trial will be treated according to the standard clinical practice of each participating center, by means of transanal endoscopic surgery.

### Recruitment and study scope

To achieve the planned sample size and enhance external validity, this study will be conducted as a multicenter trial. Spanish hospitals with specialized units in colorectal surgery, radiology, oncology, and radiotherapy will participate. Most centers belong to the TAUTEM study network [22].

Participating centers: 1. Parc Tauli University Hospital; 2. Mutua de Terrassa University Hospital; 3. Bellvitge University Hospital; 4. Joan XXIII University Hospital; 5. Vall d’Hebron University Hospital; 6. Hospital de San Juan Despí Moisès Broggi; 7. Santa Creu i Sant Pau University Hospital; 8. Donostia University Hospital: 9. Complejo Hospitalario Universitario Torrecárdenas; 10. Hospital Universitario Fundación Jiménez Díaz; 11. Hospital Clinic University Hospital; 12. La Fe University Hospital.

### Informed consent and legal considerations

The protocol, patient information, and informed consent forms will be approved by the Ethics Committees for Research with Medicinal Products (CEIm) of all participating centers. The trial is registered at ClinicalTrials.gov (ID: NCT06450574). All patients must sign the informed consent form prior to randomization.

The study will be conducted in accordance with current Spanish legislation, with authorization from the Spanish Agency for Medicines and Medical Devices (AEMPS), the 7th revision of the Declaration of Helsinki [29], the SPIRIT 2013 guidelines for clinical trials [30], and the CONSORT statement for randomized controlled studies [31].

All trial documentation (protocol and consent forms) will be processed through the CTIS (Clinical Trial Information System) portal for AEMPS authorization, in compliance with Royal Decree 1090/2015 [32]. The reference CEIm will be the CEIm Parc Taulí, which, along with AEMPS, will evaluate the methodological and clinical procedures of the trial and safeguard participant safety and welfare, reviewing all information provided to participants.

### Randomization

Patients who meet all inclusion criteria, none of the exclusion criteria, and have signed the informed consent form will be randomized. Allocation to the CRT–TEM or TEM group will be performed in a 1:1 ratio using a computer-generated randomization sequence implemented in the electronic Case Report Form (eCRF) centralized by the Contract Research Organization (CRO). The system will automatically assign treatment once eligibility criteria are confirmed.

### Study procedures (Fig. 1)

**Fig. 1:**
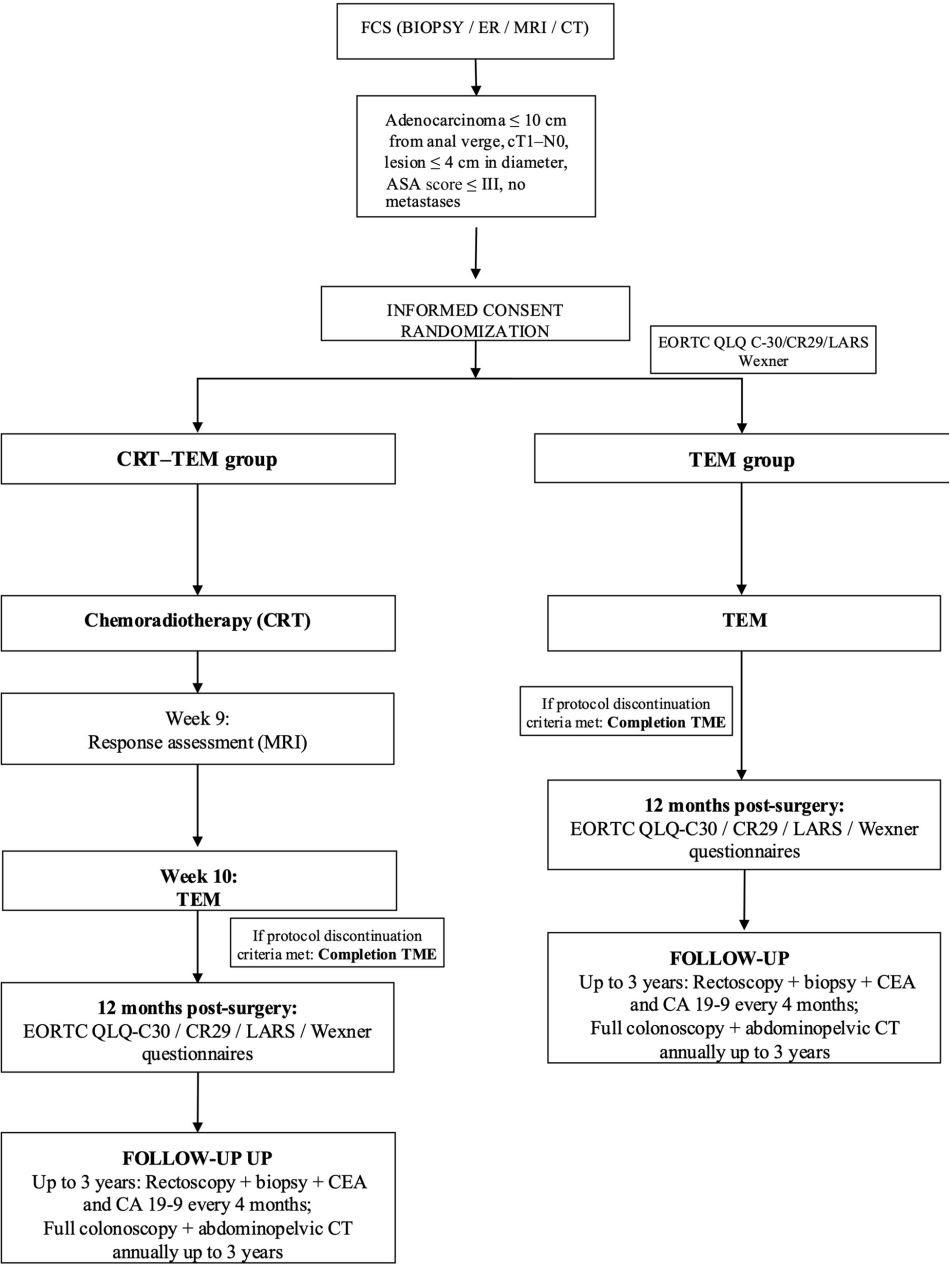
CONSORT trial flow chart of the TAUTEM-T1 study. CRT, chemoradiotherapy; TEM, transanal endoscopic microsurgery; TME, total mesorectal excision; MRI, magnetic resonance imaging; ER, endorectal ultrasound; FCS, full colorectal study; QoL, quality of life; CEA, carcinoembryonic antigen; CA 19–9, carbohydrate antigen 19–9; EORTC, European Organisation for Research and Treatment of Cancer; LARS, low anterior resection syndrome

Before TEM surgery or initiation of CRT, all patients will complete validated quality-of-life questionnaires from the European Organization for Research and Treatment of Cancer (EORTC) [33]: EORTC QLQ-C30 (version 2) [34] and EORTC QLQ-CR29 (colorectal cancer–specific module) [35]. Anorectal function will also be assessed using the LARS score [6, 36] and the Wexner fecal incontinence questionnaire [37].

### Chemoradiotherapy-TEM group (CRT-TEM)

#### Neoadjuvant treatment: preoperative CRT (long-course regimen)

Neoadjuvant chemotherapy will be administered concomitantly with radiotherapy and will consist of oral capecitabine 825 mg/m^2^ every 12 h on radiotherapy days.

Long-course radiotherapy will be administered using one of the following techniques, depending on institutional availability, with preference for the first:

- Volumetric modulated arc therapy (VMAT). Two treatment fields will be defined. Primary (standard) field includes the tumor, the mesorectum, and the regional pelvic lymph nodes, delivering a total dose of 45 Gy in 25 fractions (1.80 Gy/fraction). For the elective pelvic CTV, the cranial border will be set at the S2–S3 junction to minimize toxicity in this cT1N0M0 population. Cranial extension up to the sacral promontory/common iliac bifurcation may be considered only when clinically indicated (e.g., higher tumor location or suspicious nodal disease). Boost field: includes the tumor area and any pathological adjacent lymph nodes, delivering a total dose of 50 Gy in 25 fractions (2.00 Gy/fraction). An integrated boost technique, inherent to VMAT, will be used [38].
- Conventional three-dimensional planning (3D-CRT). The same target volumes as in (1) will be treated but using a sequential boost: 45 Gy in 25 fractions to the primary field, followed by 5.40 Gy in 3 fractions to the boost field. This schedule results in 28 treatment sessions, with the same fractionation (1.80 Gy/fraction) [39, 40].

At week 9 after completion of CRT, clinical response will be assessed by pelvic MRI, endoscopic and digital rectal exam [41]. CRT-related adverse events will be prospectively recorded and graded according to CTCAE v5.0. Acute toxicity will be collected from CRT initiation through 30 days after TEM, and late toxicity will be collected thereafter during follow-up up to 3 years.

#### Local excision by transanal endoscopic surgery

Local excision will be performed using transanal endoscopic surgery at week 10 after neoadjuvant treatment (TEM [42], TEO [43], or TAMIS [44]) under general or spinal anesthesia. Preoperative preparation will include mechanical bowel cleansing, and antibiotic and thromboembolic prophylaxis.

To ensure reliable identification of the original tumor site at week 10—particularly in cases of marked regression—baseline lesion localization will be standardized and recorded using (i) distance from the anal verge to the distal tumor margin (rigid rectoscopy and/or endoscopy) and (ii) circumferential quadrant/clock-face position (anterior, posterior, right or left lateral), together with high-quality baseline endoscopic photo documentation. Tattooing with carbon/ink is not recommended, as diffusion may hinder subsequent identification of the residual scar.

The procedure will consist of full-thickness excision centered on the scar/original tumor site, even when only a subtle scar is visible, with a circumferential margin of approximately 20 mm around the scar (minimum 10 mm), without entering the perirectal fat [7].

### TEM group (TEM)

Patients assigned to the TEM group will undergo transanal endoscopic microsurgery, following the same local excision procedure described above.

### Pathological study

Histologic tumor regression after CRT will be evaluated according to the Bouzourene classification (GR1: no residual tumor—complete regression; GR2: isolated residual tumor glands; GR3: predominance of fibrosis over tumor; GR4: predominance of tumor over fibrosis; GR5: no tumor changes—absence of regression) [45] and/or the Dworak classification (GR0: no tumor changes—no regression; GR1: dominant tumor mass with fibrosis and/or vasculopathy, regression < 25%; GR2: predominance of fibrosis with few glandular clusters—26–50%; GR3: only occasional residual glands— > 50%; GR4: no residual tumor—complete regression) [46].

The pathological report of the local excision specimen will include: lesion size (maximum and minimum diameters, in mm), adenocarcinoma differentiation grade, degree of submucosal invasion, tumor regression grade (according to Bouzourene or Dworak), pathological stage (pT1, pT2, pT3), venous, lymphatic, or perineural invasion, tumor budding, and margin status (minimum distance in mm).

The degree of submucosal invasion will be determined using the Taulí-pT1 classification described by Casalots et al. [11], based on the residual healthy submucosa (hrSB); the percentage of invasion is calculated as: [(total submucosal thickness – hrSB)/total submucosal thickness)] × 100 [11, 13]. In case of diagnostic difficulties at participating centers, samples will be sent to the Pathology Service of Parc Taulí Hospital for centralized review.

### Definition of clinical and pathological response to preoperative chemoradiotherapy

At week 9 after completion of preoperative CRT, and prior to TEM, clinical response will be assessed using a multimodal approach, integrating digital rectal examination (DRE), endoscopy, and pelvic magnetic resonance imaging (MRI).

Clinical response will be categorized as complete, near-complete, or incomplete based on the combined findings of these assessments. Complete clinical response will be defined by the absence of palpable tumor on DRE, no visible residual lesion on endoscopy (flat scar with or without telangiectasia), and no evidence of residual tumor or suspicious lymph nodes on high-resolution MRI, including diffusion-weighted imaging. Incomplete response will be defined by the presence of residual tumor on clinical, endoscopic, or radiological evaluation [41].

Regardless of response category (complete, near-complete, or incomplete), patients who remain staged as cT1N0 on week-9 MRI restaging will proceed to protocol-mandated local excision at week 10. A watch-and-wait strategy is not permitted within the trial, as histopathologic assessment of the excision specimen is required to guide the predefined escalation algorithm.

Pathological response will be classified according to Bouzourene or Dworak: pCR, Bouzourene GR1 or Dworak GR4; pPR, Bouzourene GR2–GR3 or Dworak GR2–GR3; and absence of pathological response, Bouzourene GR4–GR5 or Dworak GR0–GR1.

### Criteria for exiting the study protocol

The study protocol will be discontinued and patients will proceed to standard radical surgery with TME in the following pre-excision situations: (i) local excision is not performed using standard transanal endoscopic surgery techniques (TEM, TEO, or TAMIS) or requires conversion to abdominal surgery; or (ii) at week 9 after completion of neoadjuvant therapy, pelvic MRI restaging shows ≥ cT2 and/or cN1 (any T) [41].

Patients who do not complete neoadjuvant therapy due to toxicity, intolerance, or personal decision, and patients with an incomplete clinical response at week 9 without MRI upstaging, will still proceed to local excision (TEM/TEO/TAMIS) as per protocol; treatment modifications and response category will be recorded for secondary analyses.

Following the pathological report of the local excision specimen, the protocol will be terminated and completion TME will be indicated if any of the following are present: adverse prognostic factors (grade G3, venous, lymphatic, or perineural invasion, or tumor budding); absence of the muscularis propria layer preventing evaluation of submucosal invasion; surgical margin (lateral or deep) < 1 mm; submucosal invasion classified as sm3 according to the Taulí-pT1 classification; or pathological stage higher than pT1 or ypT1.

Absence of pathological response to CRT (Bouzourene GR4–GR5 or Dworak GR0–GR1) alone, in the setting of a pT1/ypT1 lesion with clear margins and no adverse features, will not mandate completion TME and patients will continue standard surveillance. Completion TME will be performed within 4–6 weeks according to institutional guidelines and protocols.

### Follow-up (Fig. 1)

In both groups, quality-of-life and bowel dysfunction questionnaires (EORTC QLQ-C30, EORTC QLQ-CR29, LARS, and Wexner) will be administered 12 months after surgery. For patients with a protective stoma, these assessments will be performed 12 months after stoma closure.

During the first three years, digital rectal examination and rectosigmoidoscopy with multifocal biopsies of the scar will be performed every 4 months. If any biopsy is positive for adenocarcinoma or if clinical findings are suspicious, extension studies will be completed with endorectal ultrasound (ER) and/or pelvic MRI. Tumor markers (CEA and CA 19–9) will be measured every 4 months. In addition, abdominopelvic CT and chest X-ray will be performed annually (up to 3 years). A total colonoscopy will be scheduled 1 year after surgery and, if normal, subsequent surveillance will follow each center’s institutional protocol.

### Study variables

#### Primary variable (endpoint)

Difference in rectal preservation rate between CRT–TEM and TEM at 3 years after surgery. The database will be locked after all randomized patients have completed a minimum of 3 years of follow-up for the primary endpoint assessment.

Rectal preservation failure is defined as any completion TME due to protocol discontinuation criteria (e.g., adverse pathology or inadequate response) and/or any salvage TME performed for local recurrence during follow-up.

Local recurrence will be considered a rectal preservation failure irrespective of the subsequent management strategy (including cases treated without TME). Any TME performed for any reason during the 3-year follow-up (including postoperative complications, patient preference, or conversion to abdominal surgery resulting in TME) will be classified as rectal preservation failure. A temporary diverting stoma does not constitute rectal preservation failure; however, a permanent stoma at 3 years (even in the absence of TME) will be classified as rectal preservation failure. Death before 3 years and losses to follow-up will be handled under the ITT framework; in the primary analysis, patients without documented rectal preservation status at 3 years will be conservatively counted as failures, with sensitivity analyses using alternative assumptions. Patients who do not undergo TME for any reason but have persistent/uncontrolled primary disease will be counted as rectal preservation (rectum in situ) but will be recorded as local failure and as non–disease-free (DFS event).

#### Secondary variables

CRT-related adverse events will be prospectively recorded and graded according to CTCAE v5.0 [47]. Acute toxicity will be collected from CRT initiation through 30 days after TEM, and late toxicity will be collected thereafter during follow-up up to 3 years.

*Quality-of-life outcomes* will be assessed using the EORTC QLQ-C30, EORTC QLQ-CR29, Wexner, and LARS questionnaires before surgery and at 12 months. Clinical response to CRT will be evaluated using the predefined multimodal clinical assessment.

*Demographic variables:* age, sex, and recruiting hospital.

*Preoperative or pre-neoadjuvant phase:* Baseline tumor characteristics will include tumor size (measured by colonoscopy, endorectal ultrasound [ER], and pelvic MRI; maximum diameter in mm), distance from the anal verge, tumor location (anterior, posterior, right or left lateral), percentage of rectal circumference involved, ER and MRI staging, presence of distant metastases (thoracoabdominal CT), preoperative tumor markers (CEA and CA 19–9), and histological grade on biopsy. After completion of neoadjuvant chemoradiotherapy and prior to local excision, clinical response will be assessed using a multimodal approach integrating digital rectal examination, endoscopy, and pelvic MRI, and will be categorized as complete, near-complete, or incomplete clinical response, according to predefined criteria.

*Surgical variables*: the technique employed, operative time, estimated blood loss, intraoperative complications, conversion to abdominal surgery, en bloc resection, and closure of the defect.

*Pathological variables*: tumor size, differentiation grade, response grade to CRT, pT stage, venous, lymphatic, or perineural invasion, tumor budding, local excision specimen margins (minimum lateral and deep distances, in mm), presence of muscularis propria, residual healthy submucosa/submucosal invasion, and sm classification according to Taulí-pT1.

*Postoperative morbidity:* will be classified according to the Clavien–Dindo system and also expressed using the Comprehensive Complication Index (CCI). The primary analysis will be performed at 30 days, and complications will be recorded up to 60 days to capture clinically relevant late events. Length of hospital stay, readmissions, and mortality will also be documented. Postoperative morbidity will be captured not only after local excision but also after any completion or salvage TME, using the same definitions and time windows (30 and 60 days).

*Oncologic follow-up:* LR (defined as adenocarcinoma in the biopsy of the scar or excision bed, or positive pelvic lymph nodes on puncture, assessed at 3 years), DS, OS, DFS and rectal cancer–specific survival.

### Sample size

The sample size calculation is based on the primary endpoint, rectal preservation, defined as the absence of the need for completion TME due to discontinuation criteria or local recurrence and rescue to TME. The assumptions are based on data from our group: in T1 tumors, the sm3 rate is 35–40%, with 5–10% of patients presenting adverse pathological factors; in the TAUTEM trial (T2–T3ab), the pCR rate was 44%, therefore a pCR > 55% is expected in T1 cases [22]. Assuming a two-sided test for difference in proportions with α = 0.05 and β = 0.20 (power 80%), 53 patients per group are required to detect as significant a difference between p₁ = 0.60 (TEM) and p₂ = 0.85 (CRT–TEM). Considering a 10% dropout rate, the total sample size will be 106 patients.

### Interim analysis and stopping rules

Given the superiority design, an interim analysis will be conducted approximately halfway through recruitment (after ~ 50% of patients have been enrolled). Early stopping for benefit will be considered using conservative statistical boundaries with appropriate alpha-spending, and the final significance threshold will be adjusted accordingly. Interim results will be reviewed by an independent oversight body, which will provide a recommendation to the steering committee regarding trial continuation, modification, or early termination.

### Study monitoring

Study monitoring will be performed by the Contract Research Organization (CRO). Contracting of the SCReN (Spanish Clinical Research Network) platform, supported by a grant from the Instituto de Salud Carlos III (ISCIII), will also include pharmacovigilance activities and data management.

### Statistical analysis

The principal analysis, from which the main study conclusions will be derived, will follow the intention-to-treat (ITT) approach, including all randomized patients. A per-protocol (PP) analysis will also be conducted, including patients from both groups who do not meet withdrawal criteria and who complete the study protocol. Losses to follow-up and their potential impact on outcomes will be analyzed. Pre-specified sensitivity analyses will assess the impact of missing primary endpoint data.

Quantitative variables will be described using mean and standard deviation or, when appropriate (based on the Kolmogorov–Smirnov or Shapiro–Wilk normality tests), median, interquartile range, and range. Categorical variables will be summarized as absolute numbers and percentages.

Comparisons between groups for quantitative variables will be performed using the Student’s t-test (when assumptions of normality and homoscedasticity are met); otherwise, the Mann–Whitney U test will be applied. Categorical variables will be compared using Pearson’s χ^2^ test or Fisher’s exact test, as appropriate.

Survival analyses (local recurrence, distant recurrence, and mortality) will be performed using Kaplan–Meier estimates and compared with the log-rank test.

In addition, an exploratory DFS analysis will be performed stratifying patients according to adverse histopathologic features identified in the local excision (TEM) specimen (e.g., lymphovascular invasion, poor differentiation, tumor budding, and depth of submucosal invasion), and results will be interpreted as hypothesis-generating due to limited power for subgroup analyses.

Statistical significance will be set at *p* < 0.05. When applicable, results will be presented with their 95% confidence intervals (95% CI).

## Discussion

Management of cT1N0M0 rectal adenocarcinoma remains heterogeneous and challenging, largely because adverse pathological features cannot be reliably identified preoperatively [48]. Endoscopic biopsy confirms invasive adenocarcinoma but does not provide the key risk factors required for definitive stratification, and cross-sectional imaging frequently categorizes these tumors at the T1–T2 interface with N0 status. Importantly, even in the absence of adverse features, long-term local recurrence (LR) after local excision may exceed the rates observed in our TAUTEM arm of T2–T3abN0M0 treated with neoadjuvant therapy plus TEM [22]. These limitations support an upfront strategy based on clinical staging (cT1N0M0) rather than postoperative pathology.

Against this background, TAUTEM-T1 will test whether preoperative chemoradiotherapy followed by transanal endoscopic surgery (CRT–TEM) reduces LR, avoids completion TME triggered by adverse pathology, and—crucially—increases rectal preservation without compromising oncologic safety. The trial is designed to balance the risks of overtreatment (administering CRT to patients who may do well with TEM alone) and undertreatment (exposing patients to completion or salvage TME in case of recurrence). By enrolling patients at the cT1N0M0 decision point, the protocol aligns with real-world clinical workflow.

Nevertheless, this strategy entails a clear trade-off: delivering pelvic CRT upfront in cT1N0M0 disease may overtreat a proportion of patients and can lead to acute and late pelvic toxicity. Moreover, pelvic irradiation may reduce the feasibility of using radiotherapy as a salvage option in the event of local recurrence and may complicate or preclude curative radiotherapy for other pelvic malignancies arising later in life (e.g., prostate or gynecologic cancers). In this context, short-term feasibility and safety of the same long-course CRT regimen have been previously shown in the TAU-TEM randomized trial, with high treatment compliance and no grade 4–5 CRT-related toxicity [49]. These long-term implications are part of the ethical rationale for this randomized trial and will be carefully monitored through systematic recording of acute and chronic CRT-related adverse events.

Pelvic CRT may impair bowel/anorectal function (e.g., urgency, frequency, incontinence and LARS), potentially offsetting organ-preservation benefits; therefore, function and QoL will be prospectively assessed using validated questionnaires (EORTC QLQ-C30/CR29, LARS and Wexner) at baseline and at 12 months (or 12 months after stoma closure).

Two alternative strategies may also be considered in cT1N0M0 disease. First, local excision with adjuvant (chemo)radiotherapy reserved for unfavorable pathology could reduce upfront CRT exposure; however, published evidence of (chemo)radiotherapy after local excision is associated with higher local recurrence rates than completion TME, particularly in pT1-2N0 disease, and may therefore represent a suboptimal rescue pathway when occult ≥ pT2 is identified [50, 51]. Second, a response-adapted approach randomizing complete/near-complete responders after CRT to watch-and-wait versus local excision is clinically appealing but requires robust evidence on pathological complete response rates and the safety of deferring surgery in cT1N0M0 tumors. TAUTEM-T1 is designed to generate this foundational evidence under standardized full-thickness excision and pathology review; if a high pCR rate and durable organ preservation are confirmed, a subsequent trial incorporating a watch-and-wait strategy in selected responders would be justified.

To our knowledge, no phase III multicenter randomized trial has specifically compared neoadjuvant therapy plus local excision versus standard TEM in T1N0M0 rectal cancer. Although earlier phase II studies in broader stages reported feasibility of organ preservation after neoadjuvant therapy, they did not focus on T1 disease and did not include a dedicated TEM comparator arm [52]. TAUTEM-T1 addresses this evidence gap using a superiority design and a patient-centered primary endpoint (rectal preservation), closely linked to functional outcomes and quality of life.

Methodologically, compare with the previous TAUTEM study [22, 24], the protocol incorporates current practice refinements: clinical response assessment around week 9 post-CRT and local excision at week 10 [53, 54]; standardized morbidity capture at 30 and 60 days using both Clavien–Dindo and the Comprehensive Complication Index [55]; centralized pathological review (including the Taulí-pT1 classification); and ITT-based inference with a planned interim analysis. These features enhance internal validity and the interpretability of functional outcomes alongside oncologic endpoints.

This trial could form the foundation for selecting cT1N0M0 patients with frequent complete clinical response for a future watch-and-wait strategy, contingent on confirmation of a high pathological complete response rate with local excision. Economically, the organ-preservation strategy is expected to reduce complex surgeries, hospital stays, stoma care requirements, and associated costs, thereby improving patient quality of life and healthcare resource efficiency.

Limitations include the logistical complexity of a 15-hospital multicenter trial; however, the majority of these centers participated in the original TAUTEM study, which supports homogeneity and high-quality trial execution. Recruitment timelines may be limited by these logistics, yet the smaller sample size relative to TAUTEM may mitigate delays. The superiority design allows an interim analysis with the possibility of early stopping for confirmed benefit, and methodological rigor is reinforced by centralized review. Although the Taulí-pT1 classification is relatively novel, its validated use and pathology support from Parc Taulí Hospital encourage broader adoption [11, 13]. Finally, selecting rectal preservation as the primary endpoint—clinically meaningful and closely linked to oncologic safety and quality of life—highlights the trial’s translational relevance. If positive, TAUTEM-T1 may help standardize stage I rectal cancer management around neoadjuvant therapy and local excision, reducing practice variability while optimizing oncologic, functional, and quality-of-life outcomes.

## Conclusion

This study aims to increase the rate of rectal preservation in cT1N0M0 rectal cancer through preoperative chemoradiotherapy (CRT) combined with local surgery (TEM), thereby improving oncologic outcomes—local recurrence, overall survival, and disease-free survival—as well as quality of life and bowel function.

## Data Availability

The datasets generated and/or analyzed during the current study will be made available in the Dataverse repository of the Institut d’Investigació i Innovació Parc Taulí (I3PT), the institution to which the corresponding author is affiliated. The data will be accessible upon publication.

## Declarations

### Permission to reproduce material from other sources

No permission was required to reproduce material from other sources.
